# Midfrontal conflict theta and parietal P300 are linked to a latent factor of DSM externalising disorders

**DOI:** 10.1017/pen.2023.11

**Published:** 2024-04-23

**Authors:** Phoebe S.-H. Neo, Neil McNaughton, Martin Sellbom

**Affiliations:** Department of Psychology, University of Otago, Dunedin, New Zealand

**Keywords:** Theta rhythm, P300, externalising, personality, mental disorders

## Abstract

Psychiatric illnesses form spectra rather than categories, with symptoms varying continuously across individuals, i.e., there is no clear break between health and disorder. Dimensional measures of behaviour and brain activity are promising targets for studying biological mechanisms that are common across disorders. Here, we assessed the extent to which neural measures of the sensitivity of the three biological systems in the reinforcement sensitivity theory (RST) could account for individual differences in a latent general factor estimated from symptom counts across externalising disorders (EXTs). RST explanatory power was pitted against reduced P300, a reliable indicator of externalising per previous research. We assessed 206 participants for DSM-5 EXTs (antisocial personality disorder, conduct disorder, attention-deficit/hyperactivity disorder, intermittent explosive disorder symptoms, alcohol use disorder, and cannabis use disorder). Of the final sample, 49% met diagnostic criteria for at least one of the EXTs. Electroencephalographic measures of the sensitivities of the behavioural activation system (BAS), the fight/flight/freeze system, and the behavioural inhibition system (BIS), as well as P300 were extracted from the gold bar-lemon and stop-signal tasks. As predicted, we found that low neural BIS sensitivity and low P300 were uniquely and negatively associated with our latent factor of externalising. Contrary to prediction, neural BAS/“dopamine” sensitivity was not associated with externalising. Our results provide empirical support for low BIS sensitivity and P300 as neural mechanisms common to disorders within the externalising spectrum; but, given the low N involved, future studies should seek to assess the replicability of our findings and, in particular, the differential involvement of the three RST systems.

Mental illness generates a major burden on health systems and patients (Kessler et al., 2009; Patel et al., 2018). Unfortunately, psychiatric diagnoses still rely on symptoms not causes, and so treatments remain poorly targeted (Krueger et al., 2018). Currently, clinicians diagnose disorders categorically, most recently with the Diagnostic and Statistical Manual for Mental Disorders, 5th edition, text revised (DSM-5-TR; American Psychiatric Association, 2022) and International Classification of Diseases, 11th revision (ICD-11; World Health Organization, 2021). Despite the use of symptoms as a base for diagnosis, patients with the same diagnosis may share only a few or no symptoms with each other. There is a growing consensus that such heterogeneity limits progress in understanding the neurobiological mechanisms in psychiatric disorders (Latzman & DeYoung, 2020).

A major problem for the categorical approach is that psychiatric illnesses form spectra rather than categories. Symptoms vary continuously across individuals, and there is no clear separation of health from disorder (Krueger et al., 2018, 2021; Krueger & Tackett, 2015). In spectrum models, disorders that share common features and that are frequently comorbid are clustered together (Kessler et al., 2011; Krueger et al., 2002, 2007). Notably, the features tend to form latent dimensions similar to traits in personality (Latzman et al., 2021), with chronic symptoms of disorders reflecting extremes of the traits (Latzman et al., 2021). Of particular relevance to the current study, disorders in which individuals commonly show high trait disinhibition (Mullins-Sweatt et al., 2019; Patrick, Venables et al., 2013; Venables et al., 2018) (e.g., attention-deficit/hyperactivity disorder, ADHD; substance use disorders, SUDs; conduct disorder, CD; antisocial personality disorder, APD) form an “externalising” spectrum (Kessler et al., 2011; Krueger et al., 2002, 2007). The latent factor underlying this spectrum reflects common vulnerabilities, and so, when used as a target for studying biological mechanisms, allows more precision and reliability than single categorical diagnoses (Kotov et al., 2022; Latzman & DeYoung, 2020). Since the same latent factor also appears to underlie trait disinhibition (Krueger et al., 2007; Latzman et al., 2021; Patrick, Kramer et al., 2013), in addition to clinical diagnoses of externalising disorders (EXTs), neural activity that has been associated with trait disinhibition is also implicated in externalising.

Broadly speaking, the neurobiological correlates of trait disinhibition/externalising include: (a) poor functioning in frontal-subcortical circuits (Knutson et al., 2015; Krueger et al., 2021); (b) neurotransmitter imbalances, particularly dopamine (Beauchaine et al., 2017; Pattij & Vanderschuren, 2020); and (c) reduced brain potentials, especially P300 (Bowyer et al., 2020; Krueger et al., 2021; Pasion et al., 2018; Venables et al., 2018). These correlates result from different levels and forms of analyses from multiple domains with no clear theoretical connections at the neural level and so the observed neural changes are only loosely connected with each other. Most analyses of brain functioning uses group averages and/or categorical diagnoses. Therefore, we have no clear idea of *variation* of neural activity with *variation* in individual differences in key latent factors (Latzman et al., 2021; Pasion & Barbosa, 2019). So, here, we operationalised externalising as a higher order latent factor by clustering symptoms of disorders across the externalising spectrum; and then assessed how its variation across individuals correlated with electroencephalographic (EEG) indicators of the subsystems in the revised reinforcement sensitivity theory (RST, Corr & McNaughton, 2008; Gray & McNaughton, 2000).

The RST is an extension of the state biology of animal learning, motivation, and emotion to traits as individual differences in human personality (Corr & McNaughton, 2008). RST provides a biologically detailed theoretical framework for integrating existing neuroscience with the spectrum perspectives of psychopathology (McNaughton & Corr, 2022). The subsystems (Gray & McNaughton, 2000) of RST control goal approach mediated by the behavioural activation system (BAS); goal repulsion mediated by the fight/flight/freeze system (FFFS); and goal conflict mediated by the behavioural inhibition system (BIS). Each system shows trait-like, stable patterns of reactivities, which at extreme levels are associated with psychopathology (Corr & McNaughton, 2008). BAS and FFFS are activated by cues of upcoming reward and punishment that elicit approach and active avoidance respectively. The BIS responds to conflict between concurrently activated goals, e.g., conflict between approach and avoidance generated by the co-activation of the BAS and FFFS. When goal conflict is detected, the BIS inhibits ongoing behaviours and initiates assessment of the risks of entering a potentially punishing situation by triggering scanning of the environment and memory. Resolution of the conflict is achieved by information gathering, particularly risk assessment, and by amplifying risk aversion (an increase in the strength of avoidance) until either: (a) avoidance occurs or (b) a safety signal is detected during the concurrent risk assessment.

EXT dysfunctions could result from high BAS sensitivity (and so excessive approach behaviours), low BIS sensitivity (and so failure of inhibition of inappropriate behaviours), and low FFFS sensitivity (and so failure of avoidance behaviours), or combinations of the three. Depending on the supposed underlying psychopathologies, the predicted pattern of contribution at the disorder-specific level varies (Bijttebier et al., 2009; Corr & McNaughton, 2016). However, consistent with their links to trait disinhibition, high BAS sensitivity and low BIS sensitivity are likely common factors (Corr & McNaughton, 2016).

Trait disinhibition is a broad personality construct. It includes facets of impulsivity, risk-taking, distractibility, low perfectionism, and irresponsibility (Krueger et al., 2012). Even at the facet level, the constructs are still complex and do not map onto neural mechanisms neatly (Yarkoni, 2015). With this caveat, we note that high BAS maps onto impulsivity via elevated sensitivity for reward (Gray, 1987; Smillie & Jackson, 2006). In both animal and human models of impulsivity, increasing dopamine levels reduced impulsive symptoms (Beauchaine et al., 2017; London, 2020; Pattij & Vanderschuren, 2020). On the premise that the release of dopamine is associated with reward signalling (Schultz, 2016; Schultz et al., 1997), it has been suggested that impulsive individuals engage in excessive reward-seeking in order to maintain a functional level of dopamine (Corr & McNaughton, 2016). Externalising individuals also exhibit excessive risk-taking (Krueger et al., 2012). They appear to be insensitive to the negative consequences associated with risky reward-seeking behaviours. Within the framework of RST, such insensitivity is most likely mediated by low BIS sensitivity, which leads to poor behavioural inhibition and excessive risk-taking, rather than low FFFS sensitivity, which controls behaviours when escape from punishment is the dominant goal (Corr & McNaughton, 2016; Smillie & Jackson, 2006).

A challenge in testing the RST in relation to human personality has been to separate BIS (passive avoidance, inhibition) from FFFS (active avoidance, action). The two are often confounded in extant work dominated by psychometric testing (see McNaughton & Corr, 2022; Standen et al., 2022) that subsumed FFFS in the construct of BIS, making it hard to evaluate the results on BIS–psychopathology relationships. More recently, there have been attempts to separate FFFS from BIS with questionnaires. But, care must be taken with their meaning since there is now a wide range of different RST questionnaire systems (Krupić et al., 2016) albeit with some statistical overlap (Leue et al., 2022), and none of the scales involved has ever been neurally validated.

The bedrock of RST generally and the BIS in particular is the action of selective anxiolytic drugs (Gray, 1970; Gray & McNaughton, 2000) that are not antidepressant or panicolytic. They have a consistent effect on rodent hippocampal 4–12 Hz rhythmicity (McNaughton et al., 2007), and we have shown that *all three* major chemical classes also have a consistent effect on human right frontal 4–12 Hz rhythmicity elicited by goal conflict in a stop-signal task (McNaughton et al., 2013; Shadli et al., 2015) showing that this is a biomarker of BIS sensitivity homologous to the rodent assay for anxiolytic drugs (McNaughton et al., 2007). Consistent with BIS theory, this right frontal goal-conflict-specific rhythmicity also varies with Spielberger trait anxiety (Spielberger et al., 1983) in healthy participants and among groups of anxiety disorder patients (Shadli et al., 2021).

In the context of the stop-signal task, one can apply distinct statistical contrasts to distinct times within a trial to extract rhythmicity specific not only to goal conflict but also to the conflict of outcome in contrast to intention (i.e., error, generating future avoidance, via activation of the FFFS). “For error, measurements are made locked to the timing of the mouse click. In the SST, an error occurs when a participant fails to stop clicking on a STOP trial. So, on STOP trials, a click represents a mistake, in contrast to a click in GO-trials. To extract processing specific to error, we contrasted failed STOP with successful GO trials, ignoring stop-signal delay. The majority of the EEG studies on error processing have focused on the ERN (Pasion & Barbosa, 2019). As mentioned earlier, the ERN is a response-locked potential, but is also associated with midfrontal theta rhythmic activity (Cavanagh et al., 2012; Taylor et al., 2007; Yeung et al., 2007). A Minnesota Multiphasic Personality Inventory-3 (MMPI-3) measure of the higher order spectrum of internalising psychopathology was positively correlated with action-error theta” (Neo et al., 2023).

Neural measures linked to BAS sensitivity are also available. Dopamine has been linked to approach motivation (DeYoung, 2013; Wacker & Smillie, 2015; Zisner & Beauchaine, 2015) and to high extraversion and high disinhibition (Depue & Collins, 1999; DeYoung, 2013; Smillie et al., 2019; Zisner & Beauchaine, 2015). Dopamine release is linked to signals of unexpected reward (Bromberg-Martin et al., 2010; Fiorillo, 2013; Matsumoto & Hikosaka, 2009). Reward prediction error generates an early (70–100 ms) orienting/salience component and a late (200–300 ms) motivational value component (Redgrave & Gurney, 2006; Schultz, 2016; Schultz et al., 2017) that are linked “the psychopathology-related constructs of low extraversion (social anxiety) and high disinhibition (impulsivity), respectively, making the evoked potential components biomarker candidates for indexing aberrant processing of unexpected reward” (Neo et al., 2021, p.1)

In the current study, we used our established BIS biomarker (McNaughton et al., 2013; Shadli et al., 2021; Shadli et al., 2015), together with these EEG proxies of BAS (Neo et al., 2021) and FFFS (Neo et al., 2023), to concurrently test if a latent factor of EXTs was associated with: (1) high BAS and (2) low BIS sensitivity. Critically, we tested for, but did not expect, an association with FFFS. As an active control for our latent measure of externalising, and as a contrast to the RST measures, we also tested for a negative association with P300, a brain potential that has consistently been associated with disinhibition/externalising (Bowyer et al., 2020; Krueger et al., 2021; Pasion et al., 2018; Venables et al., 2018). In doing so, we also considered whether the RST systems would increment P300 in the prediction of the latent externalising spectrum.

## 1. Methods

### 1.1. Participants

The data came from an archival database where, as part of a broader study, participants were recruited from the community with Facebook advertisements and flyers. In addition to a general advertisement looking for individuals to take part in a brain and personality study, a specific advertisement sought individuals with drug, alcohol, or anger management problems – or with ADHD or impulsivity. A total of 230 participants were tested and administered structured clinical interviews. EEG data for 206 and 160 participants were available for the stop-signal task and the gold bar-lemon task, respectively. After exclusions for EEG artefacts, and inconsistent or deviant responding detected using well-validated validity scales from the MMPI-3 (Ben-Porath & Tellegen, 2020), the sample included 144 participants in the stop-signal task (for both BIS and FFFS measures) and 137/152 participants in the gold bar-lemon task (for BAS/P300 measures). Age range and gender profile for the 206 participants consisted of 92 males, 114 females; mean age = 36; SD = 9; range = 18–56). Forty-nine percent of the final sample met diagnostic criteria for at least one DSM-5 externalising disorder: ADHD (any type, 27%); history of conduct disorder (23%); antisocial personality disorder (21%); alcohol use disorder (18%); and cannabis use disorder (12%). These rates are higher than a typical community sample (Kessler et al., 2011). This is expected because we overweighted our advert of externalising problems during recruitment. The University of Otago Ethics Committee (Health) approved our study, and the approval number is H16/031.

### 1.2. Structured clinical interviews and MMPI-3 questionnaires

We administered the Structured Clinical Interview for DSM-5 Disorders – Research Version SCID-5, First et al. (2015) and Structured Clinical Interview for DSM-5 Personality Disorders (SCID-5-PD, First et al., 2016) to assess DSM-5 EXTs. Specifically, we used the modules for attention-deficit hyperactivity disorder (ADHD), substance use disorders, intermittent explosive disorder from the SCID-5 and modules for antisocial personality disorder and conduct disorder (history) from the SCID-5-PD. Each EXT disorder was scored according to the number of criteria that were met for each disorder (i.e., count variables).

### 1.3. General EEG materials

We sampled EEG at 512 Hz referenced to CPz and recorded from FP1, FPz, FP2, F7, F3, Fz, F4, F8, T7, T8 C3, Cz, C4, P7, P3, Pz, P4, P8, M1, and M2 with an Advanced Neuro Technology (ANT) amplifier and ANT caps with AgCl electrodes. We tested and reduced impedances to below 10 KΩ with ANT software (eego) and used the Matlab 2019a plugin, EEGlab (version 2019_0) to process the data.

### 1.4. Stop-signal task (SST)

#### 1.1.1. Task description

**Figure 1.**
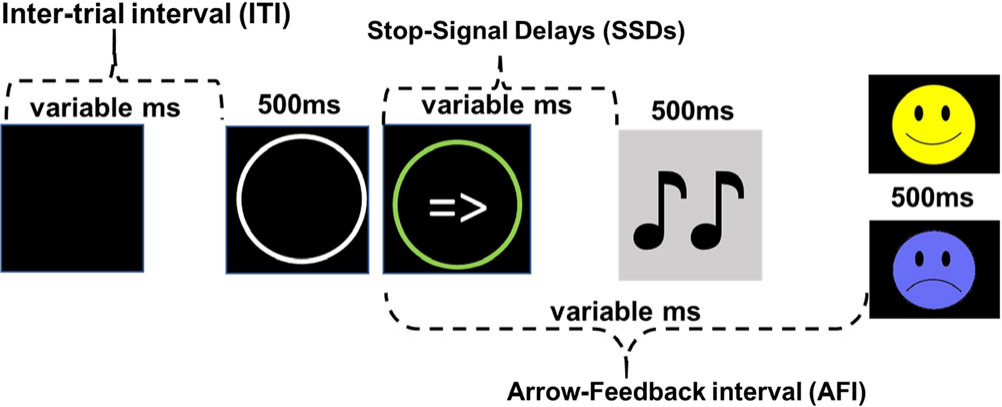
Sequence of events in a trial. Onset of the tone from the time of the arrow presentation (SSDs) in a Stop trial are variable. A Go trial follows the same event sequence but without the onset of the tone. A smiley/frowny face is presented for a successful/unsuccessful withholding of a mouse click in a Stop trial. In a Go trial, a smiley/frowny face is presented for correct/incorrect responses. ms: milliseconds. ITI: 500 ms to 4000 ms; SSDs: see Table 1; A FI: Go Correct = 1000 ms; Stop Fail = 1500 ms; Stop Correct = 1700 ms.

The stop-signal task (SST) (Logan et al., 1984), originally designed to assess motor inhibition, was modified to assess BIS goal conflict as described in (Shadli et al., 2015). Briefly, as shown in Figure 1, participants left/right mouse-clicked as quickly as possible when they see a left/right arrow (GO trials) but had to inhibit their clicking if they later hear a tone (STOP trials). On STOP trials, onset of the tone followed the arrow. The delay was determined by stop-signal delays (SSDs) controlled by separate staircases to deliver short, medium, and long SSDs. 99 STOP, and 296 GO trials were spread over three blocks (one STOP every four trials, counter-balanced in a fixed sequence). Participants practiced on 30 GO trials prior to the actual test.

#### 1.1.2. EEG processing

All recorded EEG were re-referenced (M1/M2 average) and filtered (1–36 Hz, eegfiltnew) before we applied independent component analysis (runamica15) to extract eye blink and movement components, which were then identified and removed with an automatic function (pop_icflag) using a 90% similarity threshold. Next, we extracted two 1.5 s EEG segments centred, respectively, at the stop-signal and mouse-click from each trial. Trials with EEG > ±70 µV in any sensors were excluded (pop_eegthresh). For each 1.5 s segment, we applied two consecutive 1s Hanning windows that overlapped by 0.5 s, which delivered a 1 s epoch centred at the event of interest. The Hanning window is a cosine wave that improves the quality and time resolution of Fourier transforms while having the effect of focussing power extraction on the 0.5 s at its centre. The nominal 0.5 s segment after each event was then submitted to spectral analyses, followed by log transformation to normalise the distribution. Theta power over 4–7 Hz was then averaged to deliver single power indices. Note that data from 30 participants were sampled at 256 Hz instead of 512 Hz; these were analysed as sampled.

#### 1.1.3. Neural measure of BIS tendencies

Previous work found that BIS-specific goal-conflict activity could range from 4 to 12 Hz at the right frontal sensor F8 (Neo & McNaughton, 2011; Neo et al., 2011; Neo et al., 2020; Shadli et al., 2021; Shadli et al., 2015). However, conflict activity has also been observed at the midline sensor Fz (McNaughton et al., 2013). F8 activity has been the focus of previous work because it showed consistency across studies. However, a preliminary analysis including the current sample showed that theta activity (4–7 Hz) at the midfrontal site Fz was a stronger indicator of externalising measured by MMPI-3. So here, we extracted goal-conflict-specific rhythmicity (GCSR4) using the same methods as (Shadli et al., 2015) but from Fz instead of F8. This Fz response, like the F8 one, has been shown to be sensitive to anxiolytics drugs (McNaughton et al., 2013). A STOP trial differed from a GO trial only from the onset of the tone. To extract processing specific to response inhibition (signaled by the tone), GO trial theta activity matched to the onset of the stop-signal in the adjacent STOP trial was first subtracted from the STOP trial. (Matching GO trial activity data were extracted by inserting event marks into GO trials at SSDs matched to the paired STOP trials.) On the basis that goal conflict would be high in medium SSD trials (where stopping and going were equally likely) and low in early- and late-SSDs trials, the average of early and late STOP-GO trials was subtracted from the medium trials. This subtraction controlled for non-conflict-related activity. Participants with fewer than 5 trials in any of the averages were excluded.

#### 1.1.4. Neural measure of FFFS tendencies

An error occurred when participants realised proprioceptively that they failed to inhibit a mouse-click in a STOP trial. We considered this a pure aversive signal that reflects receipt of a negative outcome, thereby eliciting avoidance tendencies controlled by the FFFS. We indexed this error-related activity with midfrontal (Fz) theta activity, which has previously been associated with errors (Cavanagh & Shackman, 2015; Cavanagh et al., 2012; McLoughlin et al., 2021; Yeung et al., 2007), and a broad range of psychopathology (McLoughlin et al., 2021). To extract processing specific to error, GO trial theta activity time-locked to a mouse click in a GO trial was subtracted from that in a STOP trial. The STOP-GO theta activity was then averaged. Individual average activity was calculated from a minimum of 20 trials.

Finally, note that the relationships between conflict and error theta activity and psychopathology (both externalising and internalising measured by MMPI-3) in the current sample were reported in Neo et al. (2023). Briefly, we found that conflict was negatively associated with externalising, and error was positively associated with internalising.

### 1.5. Gold bar-lemon task

#### 1.1.5. Task description

**Figure 2.**
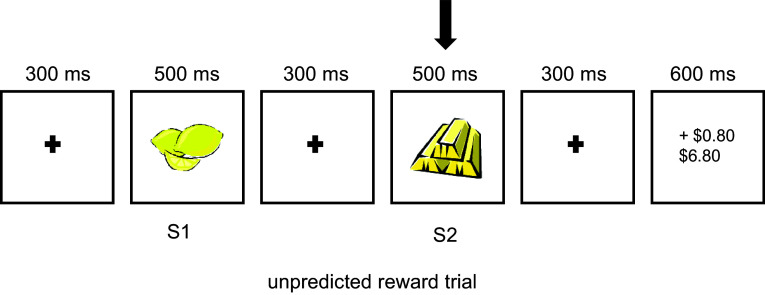
Sequence of events in an unpredicted reward trial. S1 indicates the first stimulus onset, and S2 indicates the second stimulus onset. S1 and S2 were always either a lemon or a bar. The period of interest for the current study is indicated by the vertical arrow.

Designed to test for the human homologue of dopaminergic reward-prediction-error (RPE) signalling in animal models (Potts et al., 2006), we used a version described in Neo et al. (2021). Briefly, as shown in Figure 2, two gold-bars/lemons were presented sequentially (80% of trials) and signalled expected reward ($0.50)/non-reward ($0.00). In 20% of the trials, the second cue (S2 in Figure 2) was a mismatch to the first cue (S1) and signalled unexpected reward/non-reward. 480 trials were spread over 8 blocks. Each trial was initiated by the program, and participants were told to attend to the trial closely. Although participants were informed that they would receive cash equal to the amount that they had made from the highest winnings block, the amount received was fixed at $6.

#### 1.1.6. EEG processing

All recorded EEG was filtered (eegfiltnew, lowpass at 100Hz), re-referenced to the average of all sensors other than the mastoids (M1/M2), and then segmented into epochs from S2 − 1100 through to S2 + 1400 ms. After linear drift was removed (eeglindetrend), each epoch was reduced to S2 − 100 ms through S2 + 400 ms and baseline corrected using the average of S2 − 100 ms to S2. Epochs with EEG > 70 µV in any recording sensors were rejected (pop_artextval). The epochs were than averaged according to the experimental conditions (unpredicted reward versus unpredicted non-reward trials). Participants with fewer than 20 trials in each average were excluded.

#### 1.1.7. Measure of BAS activity

**Figure 3.**
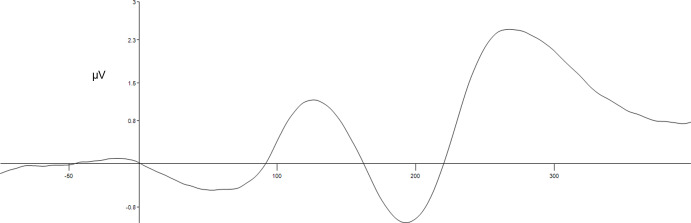
Variation of ERP across time from 100 ms before to 400 ms after the onset of S2 in the Gold bar-lemon task. The waveform shown is an average of signals recorded from P7, P3, Pz, P4, and P8. The P300 peaks at about 270 ms.

At midfrontal scalp sites, in the 200–300 ms after the onset of S2, unexpected reward trials showed a less negative potential than that of unexpected non-reward trials (Cooper et al., 2014; Smillie et al., 2011; Smillie et al., 2019; Walsh & Anderson, 2012). The difference in the potential, i.e., RPE, is thought to reflect sensitivity to dopaminergic unexpected reward processing (Neo et al., 2021). As mentioned in the introduction, dysfunctional dopamine reactivity has been theoretically linked to BAS via elevated reward sensitivity (Gray, 1987; Smillie & Jackson, 2006). So here, we use RPE signalling as a proxy of BAS activity. Specifically, we subtracted mean amplitude of Fz event-related potentials in the 200–300 ms period post S2 in the unexpected non-reward trials from those in the unexpected reward trials. Previously, we reported the relationships of the current RPE and approach motivational traits measured by MMPI-3 (Neo et al., 2021). We found that RPE was positively associated with impulsivity. The variation of the RPE signal across time, 100 ms before and 400 ms after the onset of S2, can be seen in Figure 3 of the prior study (Neo et al., 2021).

#### 1.1.8. P300

Weak “P300” reactivity is a well-established neural indicator of externalising (Bowyer et al., 2020; Pasion et al., 2018; Venables et al., 2018). The P300 is a positive brain wave observed ∼300 ms after the onset of an infrequent target stimulus. The P300 becomes larger when the target stimulus is rare. Weak P300 indicates poor attention allocation associated with disinhibition (Iacono et al., 2003). The P300 is well studied, with known neural circuitry (Polich, 2007). EEG processing of P300 here followed the same steps as those of RPE. In contrast to RPE, P300 was averaged across the unexpected non-reward and unexpected reward trials. There are two known subcomponents of P300 (Polich, 2007). Here, we averaged P300 across parietal sites (P7, P3, Pz, P4, and P8) on which the majority of the work on P300 and psychopathology has focused (Bernat et al., 2020). The averaged ERP across time is shown from 100 ms before to 400 ms after the onset of S2 in Figure 3 and shows a P300 that peaks at ∼270 ms and was assessed as the average from 200 to 300 ms to capture its upward slope.

### 1.6. General procedures

Participants first completed a battery of questionnaires for a separate study while being prepared for EEG recording. Next, they completed the stop-signal task (∼25 min, Shadli et al., 2015), followed by the gold bar/lemon task (∼30 min, Neo et al., 2021). Note that the latter task was introduced at a later stage of the study so some participants were only administered the SST. After completing the EEG recording, the participants were interviewed (see above) in a private room in the senior author’s research lab by a trained research assistant under the supervision of a registered clinical psychologist. The research assistants were clinical psychology students, with extensive practical experience, who had extensive training in these structured interviews by the senior author. They received cash winnings ($6) for the gold bar/lemon task and $50 of petrol or supermarket vouchers, as a thank you for volunteering with us. The experimental session lasted 3.5–4 h with breaks as needed.

### 1.7. Data analysis

First, we specified and estimated a one-factor EXT latent variable with the EXT disorder symptom counts as indicators. We used antisocial PD, conduct disorder, ADHD, intermittent explosive disorder symptoms, alcohol use disorder, and cannabis use disorder^1^. We used the maximum likelihood estimator with robust scaling (MLR) and treated the indicators as count data using M*plus* 8.8. Global model fit indices (confirmatory fit index, Tucker-Lewis index, root mean squared error of approximation) are not available in M*plus* with count data. Therefore, for the sole purpose of approximating model fit, we estimated a standard MLR model with symptom counts as continuous indicators.

Second, we tested a series of multiple indicators and multiple causes (MIMIC) models in which each of the EEG-based RST proxies and P300 served as predictors to determine each bivariate association with overall EXT.

Finally, we examined an MIMIC model in which all of the RST and P300 predictors were entered simultaneously to evaluate the relative incremental predictions. Full information maximum likelihood estimation was used to handle missing EEG data.

## 2. Results

Correlations among the externalising symptom counts and the neural measures used can be found in the online supplemental materials (Tables S1 and S2, respectively).

The one-factor EXT model converged appropriately. The model fit was adequate (Comparative Fit Index [CFI] = .94, Tucker-Lewis Index [TLI] = .90, Root Mean Squared Error of Approximation [RMSEA] = .098, Standardized Root Mean Square Residual [SRMR] = .055) with RMSEA likely slightly higher than expected due the small model and sample size. All factor loadings were statistically significant (*p* < .001) – unstandardised coefficients appear in Table 1.^2^ Overall, we deemed the EXT model acceptable for the primary analyses.

**Table 1.** EXT symptom count factor loadings

Symptom variable	λ	SE	*p*
Antisocial personality disorder	1.199	0.117	<.001
Conduct disorder	1.615	0.187	<.001
ADHD	0.946	0.091	<.001
Intermittent explosive disorder	2.038	0.306	<.001
Alcohol use disorder	2.037	0.332	<.001
Cannabis use disorder	2.835	0.517	<.001

We next tested our series of MIMIC models. Each of the RST constructs was added as covariates predicting the latent EXT factors. As shown in Table 2, our GCSR (BIS) and P300 variables were both significantly related to EXT in the bivariate analyses, whereas the RPE (BAS) and error (FFFS) variables were not related to EXT. In the multiple MIMIC model, all four predictors were entered simultaneously. Again, as shown in Table 2, both GCSR and P300 emerged as significant predictors, showing incremental predictive utility of EXT.

**Table 2.** Standardised coefficients from simple and multiple MIMIC models

Predictor	Bivariate MIMIC			Multiple MIMIC		
	β	SE	*p*	β	SE	*p*
RPE (BAS)	.052	.095	.584	−.021	.104	.838
GCSR (BIS)	−.209	.078	.007	−.213	.103	.039
Error (FFFS)	.145	.085	.089	.135	.101	.181
P300	−.243	.081	.003	−.229	.104	.027

## 3. Discussion

In the current study, we tested for neural mechanisms of motivational systems that are common in mental disorders within the externalising spectrum. Diagnostic symptom counts of the disorders were used to estimate a latent factor of externalising. Using RST as the framework, we made predictions on how sensitivity of each of the three neural-assessed RST sub-systems would be linked to the externalising factor (Corr & McNaughton, 2016; Gray & McNaughton, 2000). Consistent with our predictions, we found that low BIS sensitivity was, and that FFFS sensitivity was not, related to the externalising factor. Contrary to our prediction, BAS sensitivity was not associated with externalising. Consistent with existing work (Bowyer et al., 2020; Krueger et al., 2021; Pasion et al., 2018; Venables et al., 2018), we found that low P300 was also associated with externalising.

In the theory, the BIS is defined by its sensitivity to anxiolytics that are not also panicolytics. So, it can be seen as a key neurobiological system for pharmacologically defined anxiety (Gray & McNaughton, 2000). The link between BIS or low trait anxiety to EXTs was suggested more recently (Corr & McNaughton, 2016). Here, we provide empirical support for the contribution of low BIS to EXTs. Internalising and externalising psychopathology are often comorbid (Krueger & Markon, 2006) with a cross-spectrum correlation of .50. Our finding therefore begs the question of how an externalising individual with low BIS responsivity could also be, for example, anxious at the same time. It is important to note that BIS anxiety is neurologically restricted, while linguistically defined constructs of anxiety tend to encompass a wide range of “neurotic”/internalising disorders (for a discussion of the issues in mapping neuroscience and traits, see McNaughton, 2020; Yarkoni, 2015). On this view, the BIS contribution to the disorders would operate through non-shared variances between externalising and internalising. We also show here that the BIS contribution to externalising is additive to low P300. This is consistent with recent work that suggests that low P300 is linked to a common factor of internalising and externalising (Bernat et al., 2020). Such compromised brain function could include effects such as reduced stimulus significance (Hajcak & Foti, 2020) or impaired information storage (Verleger, 2020) linked to the P300 that would then add to weak conflict detection and processing by the BIS to generate disorder.

The lack of an association between externalising and high BAS may match the current state of knowledge on reward sensitivity in substance use problems, a key symptom in our estimation of the externalising factor here (Joyner et al., 2019). Different views on the nature of reward dysfunction in substance use problems have led to opposing predictions on the relationship between substance use and reward sensitivity. One view suggests that high sensitivity to reward drives gratification-seeking behaviours such as substance use (Urosevic et al., 2015). On the other hand, the reward-deficiency hypothesis suggests that low sensitivity to reward leads to substance use to make up for a low gratification baseline (Bowirrat & Oscar-Berman, 2005). The reward circuitry is complex (Ikemoto, 2010), and the different perspectives, based on necessarily reductionist views of the reward system, could reflect different aspects of the system. Furthermore, the different modules of a reward system could exert effects that interact with or cancel each other, explaining the lack of a relationship between reward sensitivity and the externalising factor here. Alternatively, it could be that reward sensitivity is disorder specific rather than being the general mechanism that we predicted. It is also worth noting that the hypothesised relationship between dopamine/reward signalling and impulsivity has primarily come from empirical work in adolescence (Beauchaine et al., 2017). Externalising in adults could have become more weighted in the prefrontal cortex as the brain matures during the developmental process (Castellanos-Ryan & Séguin, 2016). A final possibility derives from the antagonistic interactions between the positive and negative RST systems (Gray & McNaughton, 2000). In the specific form of this interaction that Corr (2001) termed the “joint subsystems hypothesis,” there is a mutual inhibition at the state level but independence of system sensitivities at the trait level. In particular, Corr (2002) found that “high impulsivity seemed to antagonise [a] BIS-mediated reaction.” Thus, in our sample, it could be that the primary effect on externalising of BIS variation would be blocked by high BAS, which would not then secondarily generate externalising itself.

Both our positive and negative results are limited by the small sample size of ∼140 where ∼250 is required for stable correlational results (Schönbrodt & Perugini, 2013). However, in practice, large sample studies, especially ones with designs like ours that require diagnostic assessments, are costly and not always feasible, and separate replication is preferable. Hence, while our results provide empirical support for low BIS sensitivity and P300 as neural mechanisms that are likely to be common across disorders within the externalising spectrum, future studies should seek to assess the replicability of our findings.

We also have not reported conventional self-report RST trait questionnaire measures since we wanted to focus solely on behavioural and electrocortical measures. Further, we believe care should be taken in interpreting such questionnaires since there is now a wide range of different RST questionnaire systems (Krupić et al., 2016) albeit with some statistical overlap (Leue et al., 2022). These involve many more dimensions than the RST theory itself (often having multiple BAS and/or FFFS scales), and none of the scales involved have ever been neutrally validated. In particular, it is worth noting that the classic so-called BIS scale (Carver & White, 1994) includes no items related to behavioural inhibition (40% involve “worry”) and is related in practice to the full range of neurotic disorders, including depression (Johnson et al., 2003), many of which are sensitive to panicolytic drugs but *not* to the anxiolytic drugs that are the key basis for defining the BIS.

## Supporting information

Neo et al. supplementary material Neo et al. supplementary material

## Footnotes

1 We considered other classes of substance use disorder, but they were deemed too infrequent (> 92% of participants met zero criteria).

2 Mplus does not generate standardized coefficients for count data. Using the suboptimal MLR estimator, standardized factor loadings ranged from .49 (Cannabis Use Disorder) to .88 (Antisocial Personality Disorder), with a median loading of .62.

## Supplementary material

The supplementary material for this article can be found at https://doi.org/10.1017/pen.2023.11.
