# Supplementary material for: Midfrontal conflict theta and parietal P300 are linked to a latent factor of DSM externalising disorders
Source: Personal Neurosci. 2024 Apr 23;7:e7. doi: 10.1017/pen.2023.11 (PMC11058520; doi:10.1017/pen.2023.11)
Supplement: Neo et al. supplementary material [file S2513988623000111sup001.docx]

**Midfrontal conflict theta and parietal P300 are linked to a latent factor of DSM externalizing disorders**

ONLINE SUPPLEMENTAL MATERIALS

Table S1

*Correlations among the externalizing disorder symptom counts.*

| Symptom variable | ASPD | CD | ADHD | IED | AUD | CUD |
| --- | --- | --- | --- | --- | --- | --- |
| Antisocial PD (ASPD) | -- |  |  |  |  |  |
| Conduct Disorder (CD) | .74 | -- |  |  |  |  |
| ADHD | .53 | .38 | -- |  |  |  |
| Intermittent Explosive Disorder (IED) | .56 | .48 | .46 | -- |  |  |
| Alcohol Use Disorder (AUD) | .47 | .36 | .25 | .35 | -- |  |
| Cannabis Use Disorder (CUD) | .38 | .36 | .28 | .36 | .51 | -- |

Table S2.

*Correlations between the neural measures.*

|  | RPE | GCSR | Error | P300 |
| --- | --- | --- | --- | --- |
| RPE |  |  |  |  |
| GCSR | .01 |  |  |  |
| Error | -.07 | -.09 |  |  |
| P300 | -.03 | .10 | .02 |  |
